# HECTOR: a parallel multistage homopolymer spectrum based error corrector for 454 sequencing data

**DOI:** 10.1186/1471-2105-15-131

**Published:** 2014-05-06

**Authors:** Adrianto Wirawan, Robert S Harris, Yongchao Liu, Bertil Schmidt, Jan Schröder

**Affiliations:** 1 Institut für Informatik, Johannes Gutenberg Universität Mainz, Mainz, Germany; 2 Department of Biology, The Pennsylvania State University, University Park, State College, Pennsylvania, PA 16801, USA; 3 Bioinformatics Division, Walter and Eliza Hall Institute, Melbourne, Australia; 4 Department of Molecular Medicine, The University of Melbourne, Melbourne, Australia

**Keywords:** NGS error correction, Homopolymer-length error, 454 sequencing, Parallelization

## Abstract

**Background:**

Current-generation sequencing technologies are able to produce low-cost, high-throughput reads. However, the produced reads are imperfect and may contain various sequencing errors. Although many error correction methods have been developed in recent years, none explicitly targets homopolymer-length errors in the 454 sequencing reads.

**Results:**

We present HECTOR, a parallel multistage *h*omopolymer spectrum based *e*rror *c*orrec*tor* for 454 sequencing data. In this algorithm, for the first time we have investigated a novel homopolymer spectrum based approach to handle homopolymer insertions or deletions, which are the dominant sequencing errors in 454 pyrosequencing reads. We have evaluated the performance of HECTOR, in terms of correction quality, runtime and parallel scalability, using both simulated and real pyrosequencing datasets. This performance has been further compared to that of Coral, a state-of-the-art error corrector which is based on multiple sequence alignment and Acacia, a recently published error corrector for amplicon pyrosequences. Our evaluations reveal that HECTOR demonstrates comparable correction quality to Coral, but runs 3.7× faster on average. In addition, HECTOR performs well even when the coverage of the dataset is low.

**Conclusion:**

Our homopolymer spectrum based approach is theoretically capable of processing arbitrary-length homopolymer-length errors, with a linear time complexity. HECTOR employs a multi-threaded design based on a master-slave computing model. Our experimental results show that HECTOR is a practical 454 pyrosequencing read error corrector which is competitive in terms of both correction quality and speed. The source code and all simulated data are available at: http://hector454.sourceforge.net.

## Background

The rapid progress of next-generation sequencing (NGS) has enabled the high throughput production of reads at low cost. The increased throughput and decreasing per base cost have made NGS an affordable tool. Many NGS sequencing technologies have been developed [1] including widely established platforms such as Illumina [2], 454 [3] and SOLiD [4] as well as newer platforms like Ion Torrent [5]. However, the reads produced are not perfect and may contain various types of sequencing errors, i.e. substitutions, insertions and deletions. These sequencing errors complicate data processing for many applications of NGS, e.g. NGS read mapping [6-9] and *de novo* genome assembly [10]. Error correction aims to identify the mistakes made by the sequencing platform by exploiting the redundancy of the reads and then correct those mistakes. Therefore, error correction to improve the sequence accuracy is an important task in bioinformatics.

Different sequencing platforms usually have different sequencing error characteristics thus complicating the error correction task. For example, while substitution errors are the dominant error source in Illumina and SOLiD, insertion and deletion (indel) errors are abundant in 454 sequencing, mainly due to homopolymers. Homopolymers are consecutive repetitions of a letter in a string. From a genomics perspective, homopolymers are sequences of identical bases, e.g. AAAA or TTT. In 454 sequencing, the homopolymer-length errors are caused by flows, which are indicated by light signals. In the base-calling step, the flow values are rounded to the nearest integer, where each flow represents a homopolymer of As, Cs, Gs or Ts and the intensity of the light determines the length of the homopolymer. Undetermined bases are represented by Ns. A homopolymer-length error occurs when the exact intensity of the light is wrongly determined. For example, a homopolymer of AAAA is determined as AAAAA, or vice versa. Subsequently, a quality score is assigned to each called base. A more detailed description is available in [3]. A study by Huse et al. [11] shows that in a typical 454 experiment, 18% of the reads have at least one error. The study also shows that insertion, deletion, substitution errors caused by homopolymers have error proportions of 20%, 9% and 10%, respectively.

Many tools for NGS read error correction use the spectral alignment method which was first introduced in the Euler-SR assembler [12,13]. The spectral alignment method establishes a spectrum of trusted *k*-mers from the input dataset and then corrects each read so that it only contains *k*-mers from the spectrum. SHREC [14], a stand-alone NGS read error correction method based on a parallelised suffix-trie with a majority voting scheme, was introduced in 2009 and was soon followed by other error correction tools, e.g. HiTEC [15], DecGPU[16], HSHREC (Hybrid-SHREC) [17], ECHO [18], Quake [19], Coral [20] and Musket [21]. The use of these error correction tools have been shown to improve downstream applications, e.g. SNP calling [19] and de novo genome assembly [14,19-21]. A study by Salzberg et. al. [10] also shows that contig sizes often increased dramatically after error correction, as much as 30-fold. Most of these tools, however, only target substitution errors, which are abundant in Illumina but not in 454 sequencing data. The only two programs that are currently capable of handling insertion and deletion errors are HSHREC [17] and Coral [21]. HSHREC is an improvement of SHREC [14], which is able to capture up to one insertion/deletion error in a read per given iteration. Coral is a multiple sequence alignment (MSA) based error correction method, which uses *k*-mers as seed and then corrects errors by constructing a consensus sequence from the multiple alignments. However, HSHREC only handles homopolymer errors of 1 bp and Coral doesn’t explicitly model homopolymer-length errors for 454 sequencing data, instead treating them as ordinary indels [1]. Identifying and correcting indels is a non-trivial task in terms of computational overhead. Exhaustive analysis of every potential indel requires a quadratic time complexity with respect to the read length and is usually dealt with using costly dynamic programming solutions (e.g. in Coral). However, the more specific error model of the 454 sequencing platform allows the error correction step to be designed more efficiently without losing sensitivity. Another type of error corrector for 454 sequencing targets amplicon pyrosequences, e.g. AmpliconNoise [22] and Acacia [23]. AmpliconNoise applies an approximate likelihood using empirically derived error distribution to remove pyrosequencing noise from reads and is very computationally intensive. Acacia is a recently published error corrector for amplicon pyrosequences that is reported to have comparable sensitivity to AmpliconNoise but is up to 2,000x faster in terms of runtime.

We present HECTOR a parallel multistage homopolymer spectrum based error corrector for 454 sequencing data. In HECTOR, for the first time we have investigated a novel homopolymer spectrum based approach in order to cope with homopolymer-length errors, which is the dominant source of sequencing errors in 454 reads. Furthermore, inspired by Musket [21], we have utilized three techniques, namely, two-sided conservative correction, one-sided aggressive correction and voting-based refinement, to form a multistage correction workflow. The performance of HECTOR is evaluated using both simulated datasets from the *Escherichia coli* (*E. coli*) genome and real datasets from the *E. coli* and *Salmonella enterica* (*S. enterica*) genomes. The performance comparison between HECTOR and Coral shows that HECTOR yields comparable correction quality to Coral, but runs 3.7× faster on average.

## Methods

**Figure 1:**
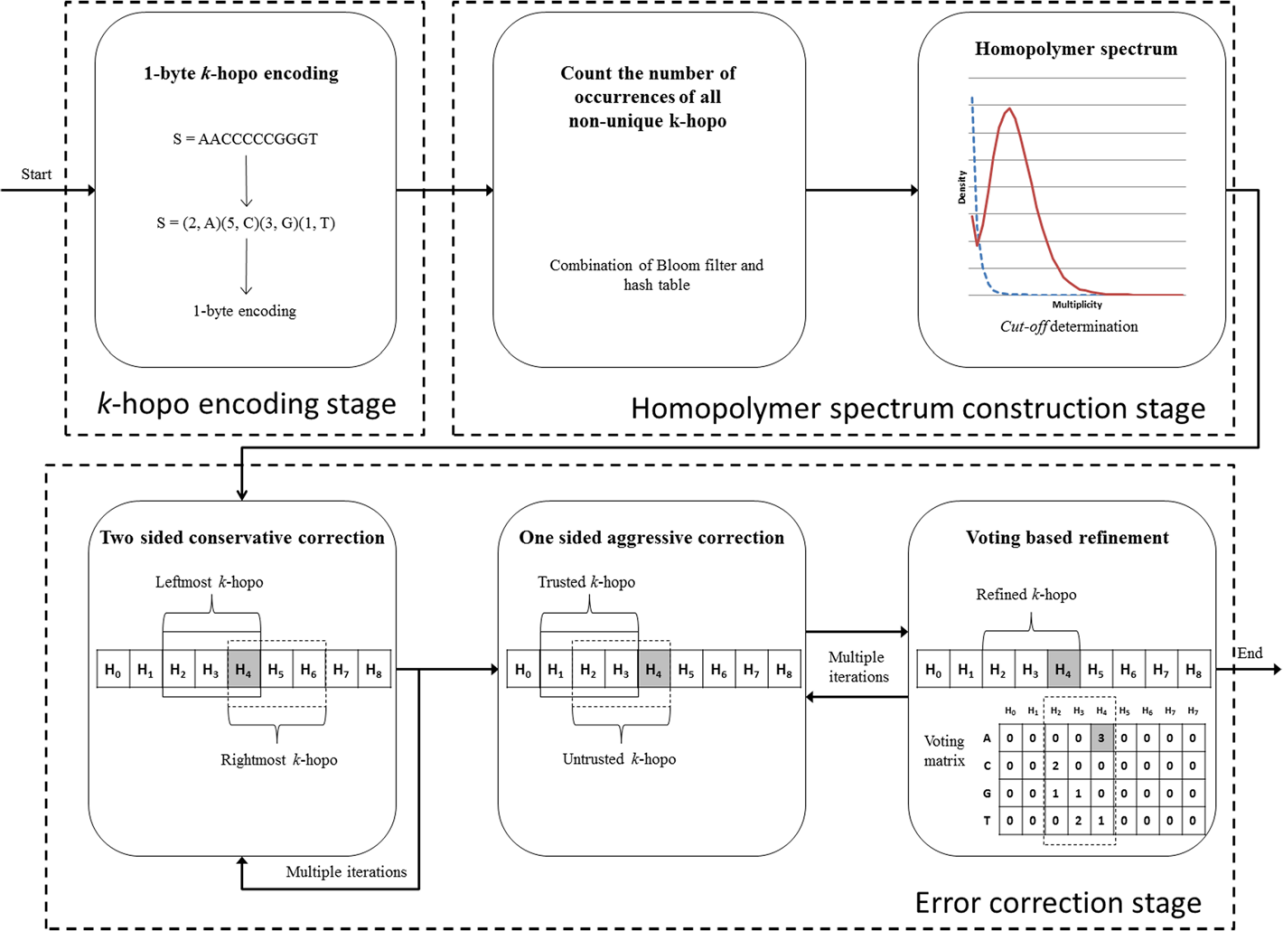
**Workflow of HECTOR.** HECTOR consists of three stages, i.e. *k*-hopo encoding stage, homopolymer spectrum construction stage and error correction stage.

As shown in Figure 1 HECTOR consists of three stages, i.e. *k*-hopo encoding stage, homopolymer spectrum construction stage and error correction stage. Firstly, HECTOR encodes a read as a string of homopolymer pairs, in which each homopolymer pair is encoded as a byte. Secondly, all of the encoded reads are then used to construct the homopolymer spectrum, where HECTOR counts the number of occurrences of all non-unique *k*-hopo using a combination of a Bloom filter [24] and a hash table. The homopolymer spectrum is defined as a set of all *k*-hopos in the dataset, where the *k*-hopos whose multiplicity exceeds a certain coverage *cut-off* are considered as trusted and otherwise, untrusted. A *k*-hopo is a substring of *k* homopolymers, in which each homopolymer is encoded as a single base. HECTOR then automatically estimates the coverage *cut-off* from the coverage histogram of all non-unique *k*-hopos. Finally, in the error correction stage, HECTOR utilizes three techniques, namely, two-sided conservative correction, one-sided aggressive correction and voting-based refinement. In addition, HECTOR takes advantage of parallelization by using multi-threading to benefit from the compute power of common multi-CPU systems. HECTOR is written in C++ and uses a combination of Pthreads and OpenMP for parallelization.

### *k*-hopo Definition and encoding

HECTOR represents a homopolymer as a pair (*R X*), where *R* is the homopolymer length and *X* ∈ Σ = {A, C, G, T, N}, and encodes a read as a string of homopolymer pairs. For example, the read *S* = AACCCCCGGGT will be encoded as (2, A) (5, C) (3, G) (1, T). In this case, all of the 3-hopos for *S* are (2, A) (5, C) (3, G) and (5, C) (3, G) (1, T). In HECTOR, we represent each homopolymer pair as a byte and then calculate the value using the following formula: (128*L + |∑|*R + *B* - 4), where *B* is an encoded nucleotide (A = 0, C = 1, G = 2, T = 3, N = 4) and *L* is the case flag (1 if the nucleotide is lowercase, 0 otherwise). Due to the 1 byte encoding implementation, the value of *R* has range between 1 and 25.

### Homopolymer spectrum construction

HECTOR encodes all reads as strings of homopolymer pairs. The homopolymer spectrum is constructed from all of the encoded reads where the counting of *k*-hopos is performed using Bloom filters and hash tables and is parallelized using multi-threading. In our implementation, the default value of *k* is 21. This value is obtained empirically and is adapted from Musket [21].

The first stage filters out as many unique *k*-hopos as possible using a Bloom filter and stores all non-unique *k*-hopos in a hash table. However some unique *k*-hopos are likely to still exist in the hash table due to the false positive probability of a Bloom filter. In this stage, the masters fetch reads in parallel from the input file and then distribute all the *k*-hopos in the reads to the slaves. The accesses to the file are mutually exclusive and are guaranteed by locks. Hashing is used to distribute each *k*-hopo to its respective destination slave to ensure a good load balance. Once the destination slave is determined, the canonical *k*-hopo is transferred to the slave through the corresponding communication channels. The canonical *k*-hopo is the smaller numerical representation of the *k*-hopo and its reverse complement. Each slave holds a local Bloom filter and a local hash table to capture all non-unique *k*-hopos as well as filter out most unique *k*-hopos. When a *k*-hopo arrives, the slave performs membership look up in the local Bloom filter for the *k*-hopos. If the *k*-hopo queried exists in the Bloom filter, the slave inserts it in the local hash table because it is likely to have more than one occurrence. Otherwise, it is inserted in the Bloom filter. At the end of this stage, all non-unique *k*-hopos are stored in all local hash tables of all slaves, and the number of unique *k*-hopos occasionally existing in all hash tables depends on the false positive probability rate of all local Bloom filters. No synchronization between the slave threads is required in this stage due to the independence of the local Bloom filters and hash tables, which greatly benefits efficiency.

The second stage computes the multiplicity of each *k*-hopo in the hash table to determine the unique hopos that are still stored in the hash table. In this stage the masters follow the same procedure as in the previous stage and all slaves listen to their corresponding communication channels and wait for the arrival of *k*-hopos. Once a *k*-hopo arrives, a slave queries the existence of the *k*-hopo in the local hash table and then increments the multiplicity if it exists. At the end of this stage, each slave holds the multiplicity of each *k*-hopo stored in its hash table, which is used to determine the uniqueness of a *k*-hopo.

The third stage removes all unique *k*-hopos from the hash table leaving all non-unique ones in place. In this stage, each slave deletes all unique *k*-hopos from its hash table while the masters are idle. At the end of this stage, each slave holds a partition of the set of all non-unique *k*-hopos in the input reads.

**Figure 2:**
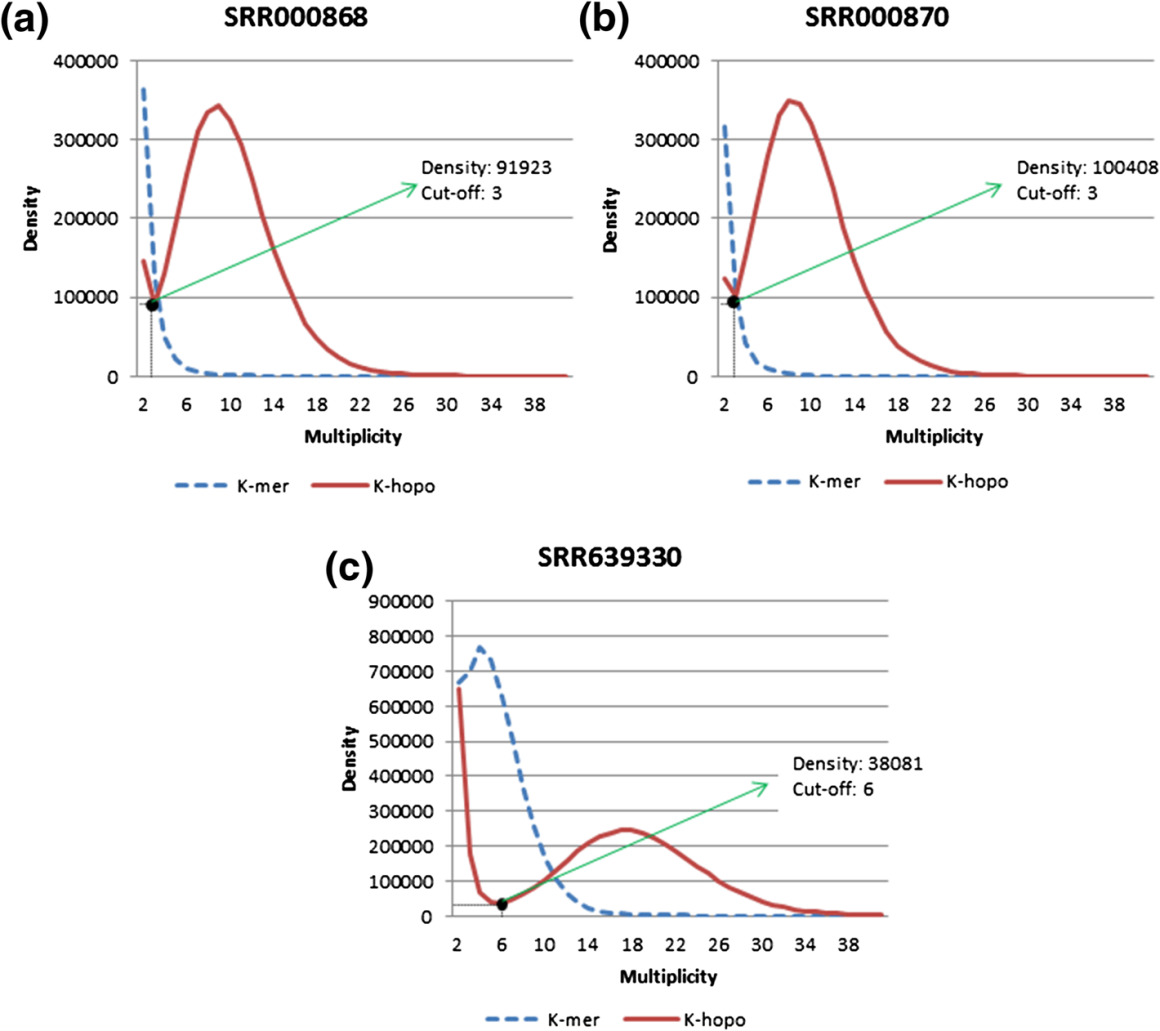
**Comparison of Homopolymer Spectrum and *k*-mer Spectrum in SRR000868 (a), SRR000870 (b) and SRR639330 (c) datasets, respectively.** A homopolymer spectrum is different from a *k*-mer spectrum, i.e. the multiplicity along the *x*-axis represents a range of actual sequence length. The comparison between the homopolymer spectrum and *k*-mer spectrum of SRR000868, SRR000870 and SRR639330 datasets are shown in **a**, **b** and **c**, respectively. The cut-off values chosen for the SRR000868, SRR000870 and SRR639330 datasets are 3, 3 and 6, respectively.

The last stage determines the coverage *cut-off* for the homopolymer spectrum from the *k*-hopo coverage histogram. A *k*-hopo coverage histogram illustrates a mixture of two distributions: one for the coverage of likely correct *k*-hopos and the other for spurious *k*-hopos. The *k*-hopos distributed at the right of the valley are supposed to be true *k*-hopos (trusted) and the ones on the opposite side to be spurious (untrusted). A homopolymer spectrum is different from a *k*-mer spectrum i.e. the multiplicity along the *x*-axis represents a range of actual sequence length. A comparison between a homopolymer spectrum and a *k*-mer spectrum of SRR000868, SRR000870 and SRR639330 reads is shown in Figure 2. In Illumina datasets where the reads have equal length, the distributions of the *k*-mer spectrum are normally bimodal. However, due to the irregular read length nature of 454 datasets, the distribution of the *k*-mer spectrum tends to be unimodal while the distribution of the homopolymer spectrum is normally bimodal. Therefore, by encoding the spectrum construction in homopolymer space, the homopolymer spectrum obtained is analogous to the *k*-mer spectrum in Musket. As seen in Figure 2a and b the homopolymer spectrums of the SRR000868 and SRR000870 datasets are similar because their datasets were sequenced in the same experiment and have similar coverage (11.7× and 11×), respectively. Figure 2c shows that the SRR639330 dataset has a different homopolymer spectrum because it has a higher coverage (69.8×) and was sequenced in a different experiment. However, in general, all the homopolymer spectrums are bimodal. HECTOR then adopts the coverage *cut-off* selection method of Musket by choosing the multiplicity corresponding to the smallest density around the valley as the coverage *cut-off*. As shown in Figure 2, the cut-off values for SRR000868, SRR000870 and SRR639330 datasets are 3, 3 and 6, respectively. These values correspond to the multiplicity associated with the smallest density around the valley in the homopolymer spectrum for each of the datasets, which are 91,923, 100,408 and 38,081, respectively. In addition, HECTOR provides a parameter to allow users to specify the *cut-off*.

### Error correction

**Figure 3:**
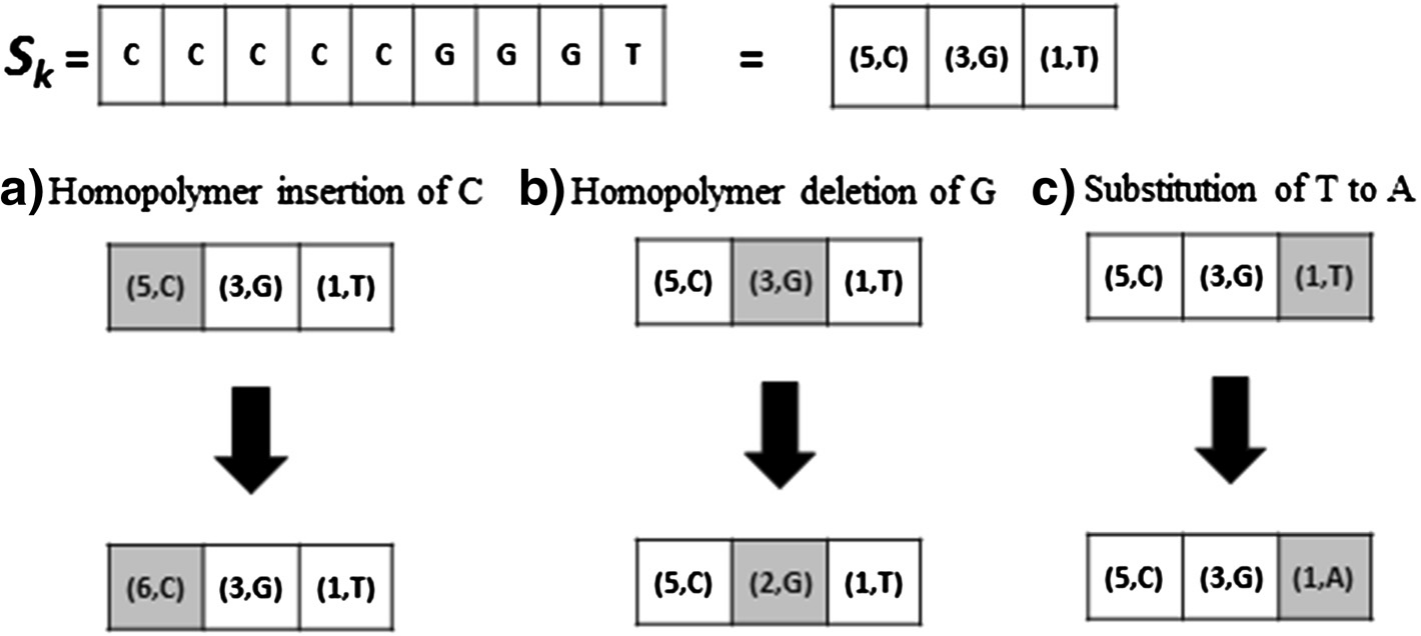
**Identification of sequencing errors in the context of k-hopos.** Illustration of how sequencing errors, i.e. insertions, deletions and substitutions, are represented in the context of k-hopos, given a k-hopo Sk. The figure shows how HECTOR handle errors in k-hopo Sk in the case of **a)** homopolymer insertion of nucleotide C, **b)** homopolymer insertion of nucleotide C, and **c)** substitution of nucleotide T with nucleotide A. Regardless of the type of error, only a single k-hopo pair is actually changed.

Figure 3 shows how sequencing errors i.e. insertions, deletions and substitutions, are represented in the context of *k*-hopos, given a *k*-hopo *S*_*k*_. Due to the representation of the *k*-hopos in (*R*, *X*) format described in the previous section, identifying a homopolymer-length error can then be represented as a difference in *R*. Therefore, whatever the type of error is, only a single *k*-hopo pair is actually changed. Thus, explaining why identification of errors in our method can be very efficient. We also ignore error corrections that occur in the first or the last homopolymer of a read. This is because the actual polynucleotide in the reference genome can be much longer than the homopolymer at the beginning or end of a read. Thus, it is impossible to tell if the read should have been longer or shorter by a single base or two.

The examples in Figure 3 are not the only conceivable scenarios of substitution and run-length errors. There are others that could change *k*-hopos more drastically. We argue in Additional file 1 why these scenarios do generally not occur in 454 data.

The error correction phase of HECTOR consists of three stages i.e. two-sided conservative correction, one-sided aggressive correction and voting-based refinement.

### Two-sided conservative correction

In the two-sided correction we assume that there is at most one error in any *k*-hopo of a read. This assumption is later relaxed in the one-sided aggressive correction. The two-sided correction starts with the classification of trusted and untrusted bases for a read. A base is considered to be trusted if it is covered by any trusted *k*-hopo. Otherwise, it is considered to be untrusted and is considered to be a potential homopolymer-length error. For a sequencing error occurring at position *i* of a read of *l* bases, it causes up to *min{k, i, l - i}* erroneous *k*- hopos. HECTOR only evaluates both the leftmost and the rightmost *k*-hopos that cover position *i* on the read, instead of all possible *k k*-hopos that cover position *i*, thus, significantly improving speed and avoiding high computational overhead. For each base, the correction is made only if an alternative is found to make both the leftmost and the rightmost *k*-hopos trusted. Otherwise, if more than one alternative is found, the base will be kept unchanged as a result of ambiguity. For a substitution error, the alternative is a homopolymer of length 1 with a different nucleotide. For an indel error, the alternative allows up to 3 bases deletion and up to 3 bases insertion of the same nucleotide. For a read, the two-sided correction will be executed for a fixed number of iterations or until no base change is made.

### One-sided aggressive correction

Since the two-sided conservative correction assumes that there is only one error in a single *k*-hopo it is inadequate in cases where more than one error occurs in a single *k*-hopo. Therefore, we utilize a one-sided correction to aggressively correct errors in these cases. The core idea is similar to the one described in [21] but we are looking for an insertion or deletion alternative, instead of a substitution alternative. Given a read *H*, define *H*_*i*_ to denote the base at position *i* of *H*, and *H*_*i,k*_ to denote the *k*-hopo starting at position *i*. If *H*_*i,k*_ is trusted, but *H*_*i+1,k*_ is untrusted, the homopolymer *H*_*i+k*_ is likely to be a sequencing error. Our one-sided correction aims to find an alternative of *H*_*i+k*_ that yields a trusted *H*_*i+1,k*_. Unlike the two-sided correction, this correction selects the alternative, which makes the resulting trusted *k*-hopo *H*_*i+1,k*_ have the largest multiplicity, if more than one alternative is found.

The one-sided correction begins with the location of trusted regions for a read. A trusted region means that every base in this region is trusted thus having a minimum length of *k*. For each trusted region, error corrections are conducted towards each of the two orientations on the read. For each orientation, the error correction process does not stop until it either reaches a neighbouring trusted region or fails to correct the current base.

This correction approach is effective but has the drawback that it strictly relies on the correctness of *k*-hopo *H*_*i,k*_. Thus if *H*_*i,k*_ does contain sequencing errors but is deemed to be trusted, this one-sided correction is possible to cause cumulative incorrect corrections, mutating a series of correct bases to incorrect ones. Look-ahead validation and voting-based refinement techniques are used to reduce this adverse effect. The look-ahead validation evaluates the trustworthiness of a predefined maximal number of neighbouring *k*-hopos that cover the base position at which a sequencing error likely occurs. If all evaluated *k*-hopos are trusted for a certain alternative on that position, this alternative is reserved as one potential correction. The voting-based refinement is described below. There is also a constraint on the number of corrections that are allowed in any *k*-hopo of a read. The default value of the constraint is 4. During the correcting process, we track the number of corrections that have been made in any *k*-hopo. If the number of corrections of a *k*-hopo exceeds the defined constraint, all corrections made in the *k*-hopo will be disregarded.

### Voting-based refinement

The voting-based refinement method used is similar to the voting algorithm originally used in DecGPU [16]. The voting algorithm attempts to find the correct base by replacing all possible bases at each position of the *k*-hopo and checking the solidities of the resulting *k*-hopos. This approach introduces the fewest new errors even though it does not correct as many errors as other correctors. However, the objective of this approach is to reduce of the number of new errors from the one-sided aggressive correction.

### Parallelization strategy

Our parallelization strategy uses the master–slave model a typical parallelization paradigm in which masters are dedicated to task distribution, and slaves are assigned to work on individual tasks. Typically in many applications, the master–slave model is implemented using a single master and multiple slaves. In HECTOR, multiple masters are used to avoid the bottleneck caused by the task distribution as the number of slaves grows larger. In general, the model is configured to have more slaves than masters, with a master-to-slave ratio chosen to be 1:3. This type of multiple masters, multiple slaves parallelization has also been implemented by Musket [21]. A hybrid combination of Pthreads and OpenMP parallel programming models is used to implement the master–slave model.

## Results and discussions

### Experimental design

The performance of HECTOR is evaluated in terms of error correction quality, runtime, and parallel scalability. All experiments were conducted on a workstation with two Intel Xeon X5650 hex-core 2.66 GHz CPUs and 96 GB RAM, running Linux (Ubuntu 12.04). Both real and simulated reads are used in the experiments. The real reads are taken from the NCBI Sequence Read Archive (SRA), i.e. SRR000868, SRR000870, SRR639330 and SRR957993. The simulated reads are generated by Mason [25], with default parameters, using *E. coli* UTI89 and *E. coli* O104:H4 as reference genomes. The performance of HECTOR is then compared to Coral (v. 1.4) and Acacia (v.1.52) using default parameters.

### Correction quality evaluations

In order to evaluate correction quality, we have to distinguish the correct from the erroneous bases in the experimental data. Errors in the sequence reads are identified by using the CUSHAW2 [9] mapping program to align reads to the reference genome. Only uniquely mapped reads with no clippings are considered, with the errors given by the differences to the reference genome. This is common practice since genomic variants such as single nucleotide polymorphisms (SNPs) can be defined as errors, and ambiguously mapped reads can lead to false classifications of bases as well [13,14,26]. However, the number of SNPs compared to sequencing errors is insignificant, so that they can be safely ignored for the purposes of experimental validation [27,28]. In addition, this is a general problem for applications like error correction, assembly, or mapping. Thus, they pose the same problem for any corrector, so that the relative results are not affected.

We have explored SHRiMP2 [29] as a potential mapping algorithm apart from CUSHAW2 and found that the reads mapped by both tools are practically equivalent. We conclude that the evaluation of error correction performance of Coral and HECTOR is independent of the mapper used to establish the gold standard of erroneous bases. For more details, please refer to Additional file 2.

The quality of the error correction is then evaluated in terms of *recall*, *specificity*, *gain*, *precision*, and *F-score*. We define *TP* (True Positive) as the number of erroneous bases that are successfully corrected, *FP* (False Positive) as the number of newly introduced errors, i.e. the number of correct bases that are changed to be erroneous, *TN* (True Negative) as the number of correct bases that remain unchanged and *FN* (False Negative) as the number of erroneous bases that remain undetected. Please note that homopolymers that fall in the start and end of reads are not counted to obtain the statistics. *Recall* is calculated as *TP/(TP + FN)*, *specificity* as *TN/(FP + TN)*, *gain* as *(TP - FP)/(TP + FN)*, *precision* as *TP/*(*TP* + *FP*) and *F*-*score* as *2* × *precision* × *recall*/(*precision* + *recall*), respectively. All the *recall*, *specificity*, *gain*, *precision*, and *F-score* values in related tables have been multiplied by 100 and all best values have been highlighted in bold.

### Evaluation on simulated datasets

The objective of the evaluation on simulated datasets is to assess the performance of HECTOR on datasets with various average read length and coverage. The simulated reads are divided into 2 groups, i.e. the shorter reads group (Group A), which has an average read length of 200 (dataset 1 – 8) and the longer reads group (Group B) which has an average read length of 400 (dataset 9 – 16). The detailed information of the simulated datasets used in the evaluation is shown in Table 1.

**Table 1:** Information of the simulated datasets, consisting of the reference genome, length of the genome, coverage, average read length and total number of reads in the dataset

**Group**	**Dataset**	**Ref genome**	**Genome length**	**Coverage**	**Average read length**	**Number of reads**
A	1	UTI89	5,065,741	5.9x	200	150,000
	2	UTI89	5,065,741	9.9x	200	250,000
	3	UTI89	5,065,741	15.8x	200	400,000
	4	UTI89	5,065,741	19.7x	200	500,000
	5	O104	5,312,586	5.6x	200	150,000
	6	O104	5,312,586	9.4x	200	250,000
	7	O104	5,312,586	15.1x	200	400,000
	8	O104	5,312,586	18.8x	200	500,000
B	9	UTI89	5,065,741	11.8x	400	150,000
	10	UTI89	5,065,741	19.7x	400	250,000
	11	UTI89	5,065,741	31.6x	400	400,000
	12	UTI89	5,065,741	39.5x	400	500,000
	13	O104	5,312,586	11.3x	400	150,000
	14	O104	5,312,586	23.5x	400	250,000
	15	O104	5,312,586	30.1x	400	400,000
	16	O104	5,312,586	37.6x	400	500,000

Table 2 shows the correction quality results of HECTOR and Coral for the simulated 454 datasets. On average, HECTOR (Coral) scores 92.84 (89.01), 99.99 (99.99), 90.62 (87.86), 97.66 (98.72) and 95.19 (93.60) in terms of *recall*, *specificity*, *gain*, *precision*, and *F-score*, respectively for group A. Furthermore in group A, HECTOR shows better sensitivity, *F*-score and gain, while Coral outperforms HECTOR in terms of precision. For group B, HECTOR (Coral) scores on average 93.16 (91.23), 99.98 (99.99), 89.89 (89.99), 96.61 (98.66) and 94.85 (94.79). Furthermore in group B, HECTOR shows better sensitivity for low coverage datasets at the expense of higher number of false positives resulting in lower gain and precision compared to Coral, while Coral outperforms HECTOR for higher coverage datasets. Overall, for the simulated datasets, HECTOR shows a consistent performance regardless of coverage while the performance of Coral improves as the coverage increases.

**Table 2:** Correction quality results of HECTOR and Coral for the simulated datasets in terms of recall, specificity, gain, precision and F-score

**Group**	**Dataset**	**Error corrector**	**Recall**	**Specificity**	**Gain**	**Precision**	**F-Score**
A	1	HECTOR	**92.81**	**99.99**	**90.62**	97.69	**95.19**
		Coral	86.06	**99.99**	85.01	**98.80**	91.99
	2	HECTOR	**92.85**	**99.99**	**90.61**	97.64	**95.19**
		Coral	89.47	**99.99**	88.54	**98.97**	93.98
	3	HECTOR	**92.85**	**99.99**	**90.57**	97.60	**95.17**
		Coral	90.18	**99.99**	89.37	**99.11**	94.43
	4	HECTOR	**92.84**	**99.99**	**90.55**	97.59	**95.16**
		Coral	90.95	**99.99**	90.13	**99.10**	94.85
	5	HECTOR	**92.82**	**99.99**	**90.71**	97.78	**95.23**
		Coral	85.63	**99.99**	84.08	**98.22**	91.50
	6	HECTOR	**92.88**	**99.99**	**90.72**	97.73	**95.24**
		Coral	89.30	**99.99**	87.89	**98.44**	93.65
	7	HECTOR	**92.86**	**99.99**	**90.62**	97.65	**95.19**
		Coral	90.11	**99.99**	88.77	**98.54**	94.13
	8	HECTOR	**92.83**	**99.99**	**90.57**	97.62	**95.17**
		Coral	90.35	**99.99**	89.06	**98.59**	94.29
B	9	HECTOR	**93.19**	**99.99**	**89.99**	96.69	**94.90**
		Coral	90.03	**99.99**	89.09	**98.98**	94.29
	10	HECTOR	**93.19**	99.98	**89.93**	96.62	**94.87**
		Coral	90.39	**99.99**	89.47	**98.99**	94.50
	11	HECTOR	**93.17**	99.98	89.89	96.60	94.85
		Coral	91.67	**99.99**	**90.78**	**99.03**	**95.21**
	12	HECTOR	**93.17**	99.98	89.90	96.61	94.86
		Coral	92.12	**99.99**	**91.28**	**99.09**	**95.48**
	13	HECTOR	**93.13**	**99.99**	**89.89**	96.63	**94.85**
		Coral	89.81	**99.99**	88.19	**98.23**	93.83
	14	HECTOR	**93.14**	99.98	89.83	96.57	94.82
		Coral	91.80	**99.99**	**90.32**	**98.41**	**94.99**
	15	HECTOR	**93.14**	99.98	89.83	96.56	94.82
		Coral	91.95	**99.99**	**90.32**	**98.26**	**95.00**
	16	HECTOR	**93.13**	99.98	89.83	96.58	94.82
		Coral	92.06	**99.99**	**90.43**	**98.26**	**95.06**

### Evaluation on real datasets

Three real 454 *E. coli* datasets and one real 454 *S. enterica* dataset are used to evaluate HECTOR and Coral. The SRR000868 and SRR000870 reads are sequenced from the *E. coli* UTI89 strain, and the SRR639330 reads from the *E. coli* O104:H4 strain. The SRR957993 reads are sequenced from the *S. enterica* subsp. enterica serovar Virchow str. CFSAN000744. For the *E. coli* datasets, the first two datasets have relatively low coverage of 11× and 11.7×, respectively and an average read length of 257, while the latter has high coverage of 69.8× and a larger average read length of 627. The *S. enterica* dataset has coverage of 20.6× and an average length of 534. Table 3 shows the detailed information of the real datasets used in the evaluation.

**Table 3:** Information of the real datasets, consisting of the reference genome, length of the genome, coverage, average read length, total number of reads in the dataset and version of the 454 platform

**Dataset**	**Ref genome**	**Length**	**Coverage**	**Average read length**	**Number of reads**	**Version**
SRR000868	E. coli UTI89	5,065,741	11.7×	257	230,517	454 GS FLX
SRR000870	E. coli UTI89	5,065,741	11.0×	257	216,458	454 GS FLX
SRR639330	E. coli O104	5,312,586	69.8×	627	591,126	454 GS FLX Titanium
SRR957993	S. enterica	4,915,960	20.6×	534	189,508	454 GS FLX

Table 4 shows the error correction quality comparison between HECTOR, Coral and Acacia for the real datasets in terms of *recall*, *specificity*, *gain*, *precision*, and *F-score*. HECTOR demonstrates comparable error correction quality to Coral in terms of all measures. Specifically, HECTOR outperforms Coral for the SRR000868 dataset, while the latter performs better for the remaining datasets. Our performance evaluation shows that HECTOR has a superior error correction quality compared to Acacia for all real datasets. However, Acacia shows better specificity.

**Table 4:** Correction quality results of HECTOR, Coral and Acacia on the real datasets in terms of recall, specificity, gain, precision and F-score

**Dataset**	**Error corrector**	**Recall**	**Specificity**	**Gain**	**Precision**	**F-Score**
SRR000868	HECTOR	**93.58**	99.96	**88.24**	**94.61**	**94.09**
	Coral	93.49	**99.99**	87.25	93.75	93.62
	Acacia	84.17	99.98	77.61	92.77	88.26
SRR000870	HECTOR	**92.11**	99.86	85.30	93.11	92.61
	Coral	92.03	**99.99**	**85.71**	**93.57**	**92.79**
	Acacia	83.48	99.98	73.42	89.24	86.27
SRR639330	HECTOR	97.33	99.41	95.54	98.20	97.76
	Coral	**99.78**	99.92	**99.52**	**99.74**	**99.76**
	Acacia	92.56	**99.98**	90.19	97.50	94.97
SRR957993	HECTOR	94.54	96.43	88.23	93.74	94.14
	Coral	**94.55**	96.87	**89.09**	**94.53**	**94.54**
	Acacia	91.79	**99.95**	85.53	93.62	92.69

### Discussion of performance on simulated and real datasets

There are several factors that cause differences in performance between real and simulated data. Firstly, the coverage in simulated data is more uniform, whereas real data tends to follow a more variable distribution. This leads to areas in the genome that are now correctable; it can also cause false corrections, because correct sequence gets erroneously classified as errors. Secondly, the error profiles of real and simulated data can be different leading to further differences in correction performance. Overall, for the simulated datasets, HECTOR shows a consistent performance regardless of coverage while the performance of Coral improves as the coverage increases. This is consistent with the performance of the real datasets, in which HECTOR is superior for the dataset with the least coverage (SRR000868) while the performance of Coral gets better as the coverage of the datasets increases. The biggest performance difference between Coral and HECTOR occurs on the SRR639330 dataset, which has an extreme coverage of almost 70 × .

### Performance analysis based on homopolymer length

**Figure 4:**
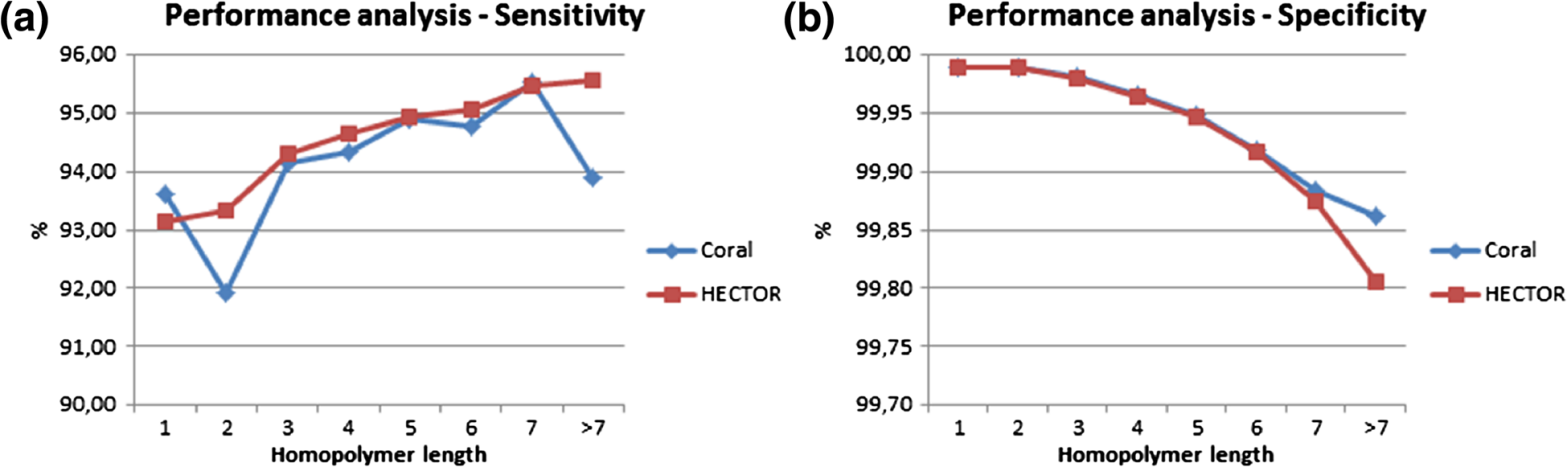
**Sensitivity and specificity of Coral and HECTOR based on the length of the homopolymer on the SRR000868 dataset. a** and **b** show the sensitivity and specificity on different length of homopolymers of both HECTOR and Coral on the SRR000868 dataset, respectively. The *x*-axis shows the homopolymer lengths of 1, 2, 3, 4, 5, 6, 7 and larger than 7, and the *y*-axis shows the sensitivity and specificity percentage, respectively.

The correction quality performance is then further analysed based on the length of homopolymers. The sensitivity and specificity of both HECTOR and Coral for homopolymer lengths of 1, 2, 3, 4, 5, 6, 7 and larger than 7 on the SRR000868 dataset are shown in Figure 4. The homopolymers are classified based on the length of the homopolymers in the original dataset. For example, if a homopolymer of length 3 gets corrected to any length, then this is classified as a correction for a homopolymer of length 3.

As shown in Figure 4a, Coral shows better sensitivity for homopolymers of length one and shows an irregular trend while the sensitivity of HECTOR improves as the homopolymer length increases. Figure 4b shows that in terms of specificity, both Coral and HECTOR show comparable performance for homopolymer length of 1, 2 and 3. As the homopolymer length gets longer, Coral shows better specificity than HECTOR. It should be noted that typically, the longer the length of the homopolymer, the less occurrence it has in the sequences. Thus, there is a higher probability of homopolymers with very long length which occur less than the required cut-off value of the homopolymer spectrum. These homopolymers will be classified as untrusted and therefore will be falsely corrected, which is one of the reasons why the specificity of HECTOR goes down as the length of the homopolymer increases.

### Run time and parallel scalability evaluations

**Figure 5:**
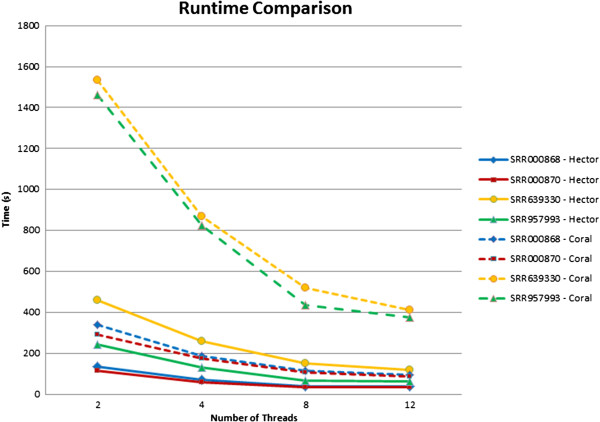
**Runtime comparison between HECTOR and Coral.** Runtime comparison of both HECTOR and Coral on the real datasets using 2, 4, 8 and 12 threads. The *x*-axis shows the number of threads, and the *y*-axis shows the runtime in seconds. HECTOR is superior to Coral for all cases. On average, HECTOR is about 3.7× faster than Coral.

In addition to correction quality, runtime and parallel scalability are important factors that must be taken into account, especially for large-scale datasets. We have evaluated the runtime and parallel scalability of both HECTOR and Coral using the aforementioned real datasets using 2, 4, 8 and 12 threads. Runtimes are measured in wall clock time. Figure 5 shows the runtime comparison between HECTOR and Coral. HECTOR is superior to Coral for all cases. On average, HECTOR is about 3.7× faster than Coral.

The run times of HECTOR are vastly superior compared to Acacia. For the SRR000868, SRR000870, SRR639330 and SRR957993 datasets, Acacia has a run time of 25, 24, 185 and 22 hours, respectively. In comparison, HECTOR has a run time of 136.9, 114.6, 459.7 and 231.3 seconds, respectively, using 2 threads. The run time of Acacia is not shown in Figure 5 because of the huge run time difference compared to Coral and HECTOR. In addition, Acacia is also only a sequential program, so it is not possible to measure the parallel run time with multiple threads.

**Figure 6:**
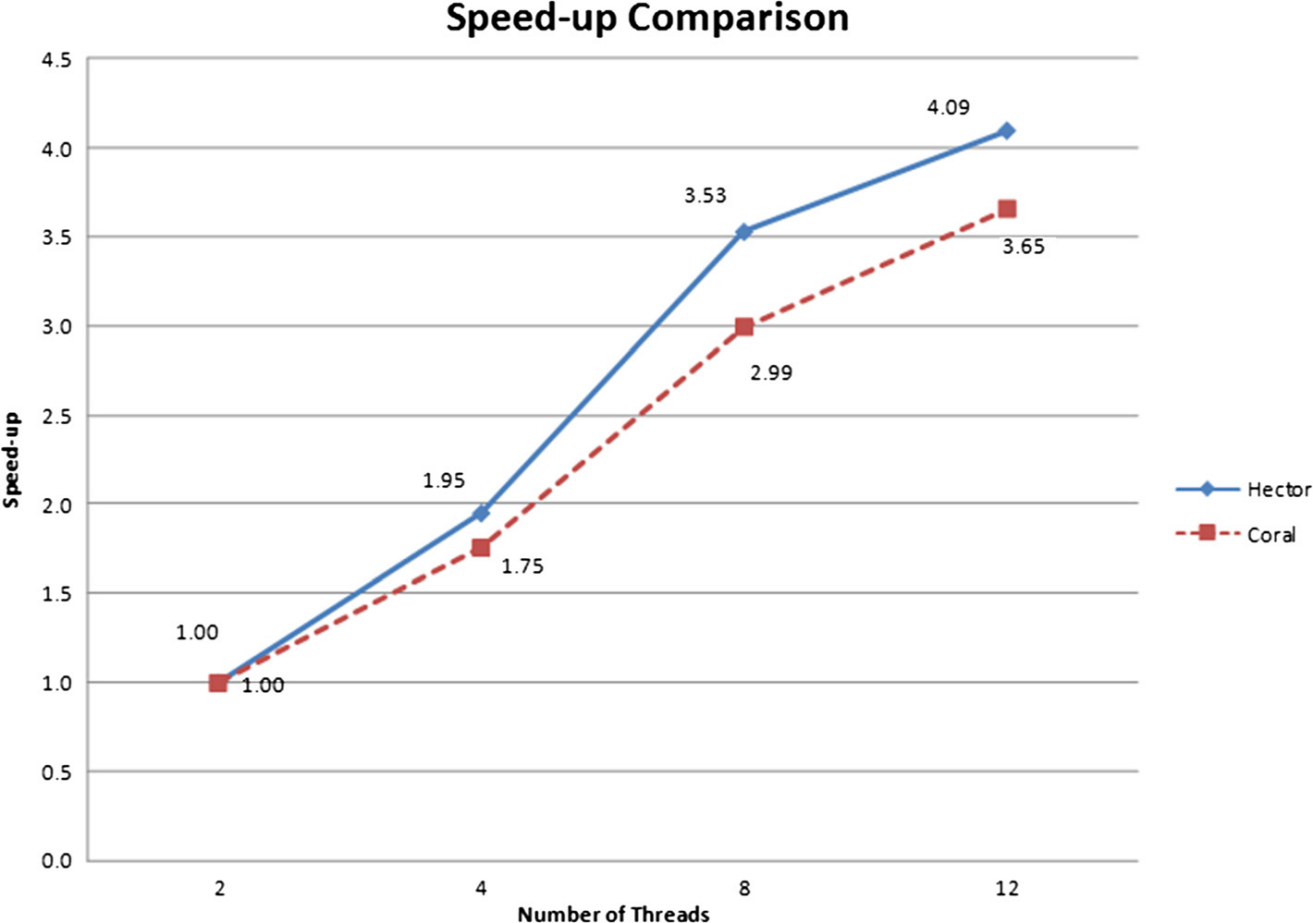
**Speed-up comparison between HECTOR and Coral.** Speed-up comparison of both HECTOR and Coral on the real datasets using 2, 4, 8 and 12 threads. The *x*-axis shows the number of threads, and the *y*-axis shows the speed-up gained. HECTOR showed a better parallel scalability compared to Coral.

The parallel scalability of HECTOR and Coral are measured by varying the number of CPU threads used. As HECTOR employs a master–slave model, all of its speed-ups are calculated against the runtime with two threads. Figure 6 illustrates the speedups of HECTOR and Coral with different number of threads. HECTOR showed a better parallel scalability compared to Coral.

## Conclusion

The current-generation sequencing technologies are able to produce low cost, high throughput reads. However, the reads produced are imperfect and may contain various sequencing errors. Error correction tools have been shown to produce better quality data, which in turn enables downstream applications to have better results, compared to without any corrections [10]. Although many error correction methods have been developed in recent years, none of them explicitly targets homopolymer-length errors in the 454 sequencing reads. We have addressed this challenge by presenting HECTOR, a parallel multistage homopolymer spectrum based error corrector for 454 sequencing data. HECTOR is based on a novel homopolymer spectrum based approach, which to the best of our knowledge is the first algorithm that can deal with arbitrary-length homopolymer indels, while maintaining a linear time complexity. Based on our *k*-hopo coding scheme, three correction techniques have been adapted: two-sided conservative correction, one-sided aggressive correction and voting-based refinement to form a multistage correction workflow. In addition, HECTOR uses multi-threading, based on a master–slave model, to leverage the compute power of common shared-memory multi-CPU platforms.

We have evaluated the correction performance of HECTOR in comparison to Coral and Acacia. The evaluations have been conducted using both simulated and real reads. For both the shorter and longer simulated reads, HECTOR achieves consistent and competitive scores in terms of the five metrics: *recall*, *specificity*, *gain*, *precision*, and *F-score*. For the real datasets, the correction quality of HECTOR and Coral are comparable in terms of all measures. HECTOR also shows a superior error correction quality compared to Acacia for all real datasets, while Acacia shows better specificity. In addition, HECTOR performs well even when the coverage of the dataset is low. On average, HECTOR runs about 3.7× faster than Coral, while demonstrating a superior parallel scalability. The run times of HECTOR are vastly superior compared to Acacia. Thus, although HECTOR is not vastly superior compared to Coral, it offers a novel approach to correct sequencing errors with competitive correction abilities and improved runtime performance.

For our future work, we are interested to see how HECTOR can improve other NGS applications, e.g. *de novo* assemblers. Examples of assemblers for 454 NGS reads include CABOG [28] and Newbler [3]. Furthermore, a recent publication [30] shows that correcting errors of 454 reads prior to transcriptome assembly can improve the *de novo* assembly process. In addition, Ion Torrent and 454 sequencing have similar error characteristics, i.e. indels are abundant due to homopolymer errors [31,32]. Therefore, it would also be interesting to extend HECTOR to correct Ion Torrent reads.

## Abbreviations

CPU: Central processing unit; NGS: Next-generation sequencing; SNP: Single nucleotide polymorphism; MSA: Multiple sequence alignment; TP: True positive; FP: False positive; TN: True negative; FN: False negative.

## Competing interests

The authors declare that they have no competing interests.

## Authors’ contributions

AW programmed the algorithm, performed the tests, analysed the results and drafted the manuscript; RH conceived the initial idea, programmed the algorithm and revised the manuscript; LY programmed the algorithm and revised the manuscript; BS analysed the results and revised the manuscript; JS analysed the results and revised the manuscript. All authors have read and approved the final manuscript.

## Supplementary Material

Additional file 1 HECTOR is capable of handling error scenarios that change more than one hopo, i.e. substitution to neighbouring hopo run, substitution of a single base/absorption in hopo run and carry forward/incomplete substitutions. Click here for file

Additional file 2 **SHRiMP2 was explored as a potential mapping algorithm apart from CUSHAW2.** The results showed that the reads mapped by both tools are practically equivalent. Click here for file
