# Supplementary material for: HECTOR: a parallel multistage homopolymer spectrum based error corrector for 454 sequencing data
Source: BMC Bioinformatics. 2014 May 6;15:131. doi: 10.1186/1471-2105-15-131 (PMC4023493; doi:10.1186/1471-2105-15-131)
Supplement: Additional file 1 — HECTOR is capable of handling error scenarios that change more than one hopo, i.e. substitution to neighbouring hopo run, substitution of a single base/absorption in hopo run and carry forward/incomplete substitutions. [file 1471-2105-15-131-S1.pdf]

HECTOR: A parallel multistage homopolymer spectrum based error corrector for 454 sequencing data

Supplementary File 1

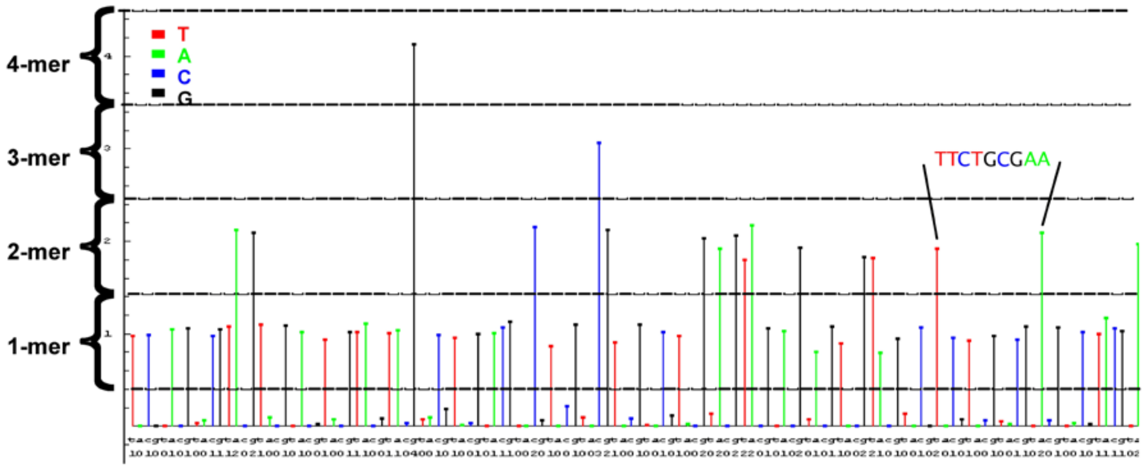

Figure S1: Example of a 454 flo-gram (illustration taken form Marguiles et al. 2005)

Error scenarios that change more than one hopo:

1. Substitution to neighbouring hopo run.
   Figure S2 illustrates this scenario. Although this scenario can in fact occur, it does not due to a substitution, but to two errors in hopo run-lengths. The example shows an example flo-gram to highlight how this can occur. Although this does lead to two changes of hopos, HECTOR can retrieve the original sequence with one sided aggressive correction

2. Substitution of a single base/absorption in hopo run.
   This scenario is more damaging to HECTOR's ability to fully correct a read: HECTOR can only modify existing hopos, it cannot create new ones, or remove others entirely. However, Figure S3 illustrates how a flo-gram for such a case would look like in two examples. Neither constellation would get past the base caller, since it would be forced to call the same base twice in a row (b), or call no base at all for at least 4 cycles (a) – both cases are impossible due to the chemistry of the sequencing platform.

3. Carry forward/incomplete substitutions
   This scenario is shown in Figure S4. HECTOR can correct this type of error by correcting the two subsequent substitution errors. In Figure S4, the error corrections made are A $\rightarrow$ G and G $\rightarrow$ A. HECTOR can correct this scenario with the one sided aggressive correction, although it leads to two changes of hopos.

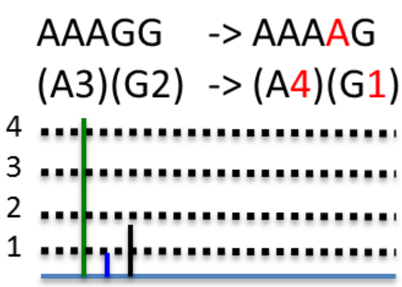

Figure S2

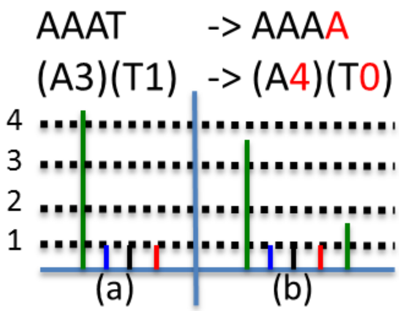

Figure S3

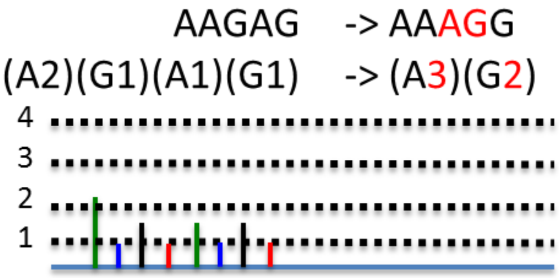

Figure S4
