# Supplementary material for: HECTOR: a parallel multistage homopolymer spectrum based error corrector for 454 sequencing data
Source: BMC Bioinformatics. 2014 May 6;15:131. doi: 10.1186/1471-2105-15-131 (PMC4023493; doi:10.1186/1471-2105-15-131)
Supplement: Additional file 2 — SHRiMP2 was explored as a potential mapping algorithm apart from CUSHAW2. The results showed that the reads mapped by both tools are practically equivalent. [file 1471-2105-15-131-S2.pdf]

HECTOR: A parallel multistage homopolymer spectrum based error corrector for 454 sequencing data

Supplementary File 2

We have explored SHRiMP2 as a potential mapping algorithm apart from CUSHAW2 and found that the reads mapped by both tools are practically equivalent.

In our experiment, we compared the performance of CUSHAW2 (v.2.4.3) against SHRiMP2 (v.2.2.3) - 64 bit for the SRR000868 dataset using default parameters. We measure how many reads are mapped before error correction and after error correction using Coral and HECTOR, respectively. As shown in Table S1, there are more reads that can be mapped using CUSHAW2 than SHRiMP2. Furthermore, error correction is shown to improve the number of reads that can be mapped by both CUSHAW2 and SHRiMP2.

Table S1. Performance comparison between CUSHAW2 and SHRiMP2

	Before error correction		Coral		HECTOR	
	Mapped	%	Mapped	%	Mapped	%
SHRiMP2	195,743	84.91	195,945	85.00	195,859	84.97%
CUSHAW2	199,637	86,60	199,776	86.66	199,713	86,64%

In addition, we look at the similarity and difference in the mapping results of both CUSHAW2 and SHRiMP2, as shown in Table S2. We consider every read that maps to the same loci with both tools as identical mapping and every read that maps to different loci as misclassification. We also only consider reads that don't contain soft clipping in concordance with our experimental design to evaluate error correctors. As shown in Table S2, the majority of the reads are identically mapped, the choice of mapper have a negligible impact on the statistical analysis of errors. To expand on this point: Only about 1% of the reads get placed differently by the two mappers – and this is an upper bound for any effect on the statistical analysis of error correction: Differently mapped reads result only *potentially* in different classification of bases as errors; in case of repeats the aligned to reference sequences might in fact be identical. We therefore conclude that the evaluation of error correction performance of Coral and HECTOR is independent of the mapper used to establish the gold standard of erroneous bases.

Table S2. Similarity and difference in read mapping between CUSHAW2 and SHRiMP2

	Identical mapping	Misclassification
Before error correction	119,413	1,545
Coral	155,022	1,556
HECTOR	137,275	1,576
